# Advancing equity in human genomics through tissue-specific multi-ancestry molecular data

**DOI:** 10.1016/j.xgen.2023.100485

**Published:** 2024-01-24

**Authors:** Ana Luiza Arruda, Andrew P. Morris, Eleftheria Zeggini

**Affiliations:** 1 Institute of Translational Genomics, Helmholtz Munich, 85764 Neuherberg, Germany; 2 Munich School for Data Science, Helmholtz Munich, 85764 Neuherberg, Germany; 3 Technical University of Munich, School of Medicine, Graduate School of Experimental Medicine, 81675 Munich, Germany; 4 Centre for Genetics and Genomics Versus Arthritis, Centre for Musculoskeletal Research, The University of Manchester, Manchester M13 9PT, UK; 5 TUM School of Medicine, Technical University Munich and Klinikum Rechts der Isar, 81675 Munich, Germany

## Abstract

There is a pressing need to generate molecular data from diverse tissues across global populations. These currently missing data are necessary to resolve genome-wide association study loci, identify effector genes, and move the translational genomics needle beyond European-ancestry individuals and the minority of diseases for which blood is the relevant tissue.

## Main text

### Genetic diversity of global populations

Within a population, the prevalence of a complex disease or the distribution of a quantitative trait is determined by the interplay of genetic and environmental factors. Understanding genetic diversity across global populations can help elucidate genetic components and underlying biological mechanisms of complex diseases/traits that traverse all populations—or are population specific. A more comprehensive representation of genetic diversity enables the development of precision-medicine approaches that address the needs of everyone, irrespective of their ancestry, ultimately leading to more equitable and democratized access to advances in healthcare.

### Diversifying genome-wide association studies

**Figure 1:**
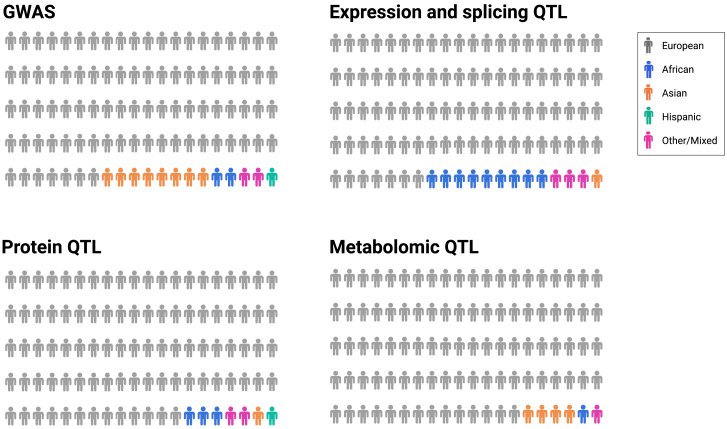
Distribution of sample ancestry in genetics and molecular studies An illustration of the ancestral composition of samples sourced from publicly available datasets across diverse genetic data modalities. Ancestry is interpreted in a broad context. The first and second quadrants present the ancestry distribution of GWAS and gene expression (RNA sequencing [RNA-seq]) data. In the upper-left quadrant, the pictogram portrays the ancestry distribution of all samples within the GWAS catalog. The GWAS sample ancestry information is derived from the GWAS Diversity Monitor (accessed on July 18, 2023), which aggregates data from the GWAS Catalog. The upper-right quadrant depicts the ancestry distribution of the available gene expression RNA-seq data from the eQTL catalog release 6, which was extracted from the official GitHub repository containing various resources related to the catalog data summary page (https://github.com/eQTL-Catalogue). The authors assigned each sample from the contributing studies to a super-population from the 1000 Genomes Project. The third and fourth quadrants display the ancestry distribution for proteomics data in the lower-left quadrant and the ancestry distribution for metabolomics data in the lower-right quadrant. Both omics modalities’ data are sourced from https://www.metabolomix.com/ (accessed on July 20, 2023).

Genome-wide association studies (GWASs) have revolutionized our understanding of the genetic basis of complex diseases/traits. GWASs are biased toward European-ancestry individuals (Figure 1). This is also the case for collections in countries with genetically diverse populations, such as the United States and United Kingdom, in which biobanks are generally not truly representative of the general population. A large part of this is due to a lack of engagement by the scientific community with diverse communities. A further reason is mistrust by these underrepresented communities due to actual and perceived bias and past harm inflicted by the medical and scientific communities.^1^ This Eurocentric bias limits the generalizability of GWAS findings and fails to capture the full spectrum of genetic variation present across global populations.^2^ For instance, African-ancestry populations have a higher level of genetic diversity compared to European- and Asian-ancestry populations, resulting in enhanced novel genetic findings per individual.

In recent years, large international collaborative efforts have been made to expand GWASs beyond European-ancestry populations.^3^^,^^4^^,^^5^ By expanding GWAS efforts to encompass individuals from diverse ancestries, novel genetic variants associated with complex traits have been identified and population-specific genetic architectures have been unraveled, shedding light on biological pathways that may underlie disease susceptibility. For example, a GWAS of estimated glomerular filtration rate (eGFR), a measure of kidney function used to define chronic kidney disease, from the Ugandan population (n = 3,288) identified a novel association at the *GATM* locus.^6^ This association is driven by an African-specific intergenic variant, which is monomorphic in European-ancestry populations and rare in East Asian-ancestry populations.

To fully understand the biological mechanisms underlying these signals and unlock the translational potential of GWAS findings, it is essential to identify the causal variants and their target genes. Fine-mapping aims to narrow down the set of potential causal variants within a given genomic region by leveraging linkage disequilibrium (LD) patterns and functional annotations. This approach benefits from data from diverse ancestries, especially those with finer-grained LD structure as compared with European-ancestry populations. The lower correlation between variant genotypes allows for a better resolution in localizing causal risk variants.

### Unraveling the translational potential: Integrating GWAS signals with molecular data

The majority of GWAS signals are located in non-coding regions of the genome, which makes it challenging to decipher their functional impact. Integrating GWAS findings with molecular data, such as gene and protein expression and chromatin accessibility and conformation, can help to overcome this challenge. Integration of GWASs with molecular data can be executed, for instance, through comparing fine-mapped datasets, colocalization analysis, and causal inference analysis using Mendelian randomization. Colocalization analysis assesses whether a GWAS signal and a molecular quantitative trait locus (QTL) signal originate from the same causal variant. A molecular QTL refers to a genomic variant that is associated with variation of a molecular trait, such as gene expression levels, protein abundance, or metabolite concentrations.^7^ By examining the overlap between GWAS signals and molecular QTLs, candidate effector genes can be prioritized. Finally, Mendelian randomization provides a framework for inferring causal relationships between genetic variants, intermediate molecular traits, and complex traits, offering insights into mechanism of action.

Despite the enormous ongoing efforts in the GWAS community to generate genetic data from diverse ancestries, a significant gap remains in the availability of molecular data from primary tissues and cells of diverse populations, coupled with genotype information^8^ (Figure 1). Returning to the novel association reported in the Ugandan GWAS of eGFR, attempts to elucidate the function of the identified intergenic variant through colocalization are limited because this variant is absent or rare in European-centric molecular QTL resources.^6^

The majority of available omics data with corresponding genetics data from Asian ancestry comes from East Asia (including Japan and China) rather than South Asia. Similarly, data from African American research participants are often reported as broad African ancestry without considering the plurality of genomic diversity in this continent. A more fine-grained definition of sample ancestry is lacking from multiple resources and might disguise the real populations where data collection efforts need to be concentrated to fill the gaps.

### Pressing demand to generate tissue-specific molecular data from diverse ancestry groups

Gene regulation is a complex process that varies across tissues and ancestries. Different cell types exhibit distinct gene expression profiles and regulatory landscapes, influencing the interpretation of GWAS results. To fully exploit the potential of large-scale genetic data, elucidating tissue-specific regulatory networks underlying complex traits, it is necessary to incorporate molecular information from the relevant primary tissue or cells of interest. Therefore, it is crucial to prioritize and invest in the collection of molecular data from a wide range of primary tissues across diverse populations to enhance our understanding of the functional consequences of genetic variation that may be specific to certain populations and pave the way for more inclusive and personalized healthcare approaches. Recently, large initiatives that include genetic data from diverse populations, such as the UK Biobank and the Trans-Omics for Precision Medicine (TOPMed) program, have included proteomics and metabolomics data from blood.^9^^,^^10^

**Figure 2:**
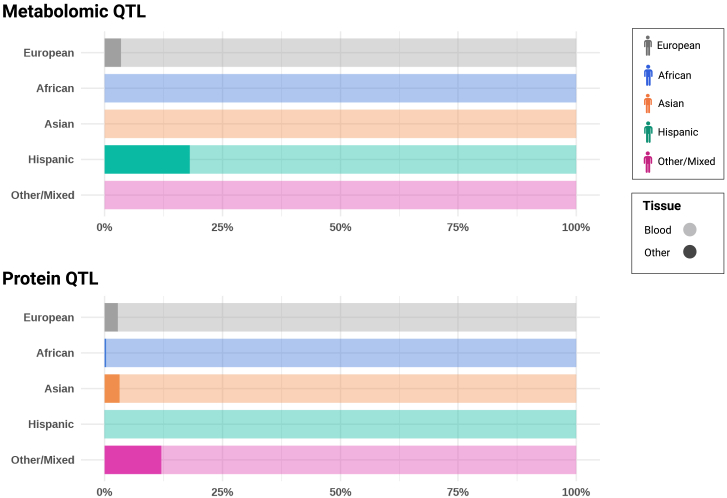
Tissue partitioning of omics samples per ancestry This stacked bar plot provides an overview of sample distribution across different tissues based on broad ancestral backgrounds. Considering that the vast majority of samples comes from blood, tissues were classified in a binary way: “blood” and “other.” For both omics modalities, data were extracted from https://www.metabolomix.com/ (accessed on July 20, 2023).

It is important to note here that most existing molecular data from diverse populations is predominantly from blood samples (Figure 2). However, different tissues in the human body can have distinct gene regulation patterns. To understand the full range of human genetics, data from primary tissues involved in complex traits are essential.^11^ Addressing this need, the ENCODE (Encyclopedia of DNA Elements) and Roadmap Epigenomics projects are significant endeavors in genetics research.^12^^,^^13^ The projects provide a systematic map and characterization of functional elements and the epigenetic landscape of the human genome, respectively. However, ancestry information for the tissue-specific molecular data is not available, hindering its integration with GWAS results to disentangle population-specific biological mechanisms.

One example of a genetic data resource that contains tissue-specific molecular data from diverse ancestry groups is GTEx.^14^ The GTEx v8 release includes whole-genome sequencing and gene expression data for 838 individuals, including 103 African American and 12 Asian American individuals from self-reported ancestry. However, when stratifying the samples by tissues, population-specific sample sizes remain low and insufficiently powered to allow the confident characterization of robust molecular QTLs in their majority.^14^ For single-cell-resolution data, the global initiative Human Cell Atlas is creating a reference map of all human cells and inclusively collecting data from populations across the globe to better represent the genetic landscape of human diversity.^15^

### Challenges and future directions

In addition to the growing drive to expand GWASs to underrepresented ancestry groups, there is now a pressing need to generate molecular data particularly from primary tissues from more diverse populations. To truly advance equity in human genomics, a multifaceted approach is crucial. This entails the allocation of funding specifically aimed at closing the gaps in genomic research and ensuring equitable distribution of resources. Intensification of efforts toward the generation of diverse-population molecular data from primary tissues, the development of robust infrastructure for sample collection, and thoughtful engagement of underrepresented communities is warranted going forward. Finally, fostering global collaboration will continue to be of paramount importance, facilitating the exchange of resources, knowledge, and data.

The integration of genetic and molecular data across populations requires sophisticated computational tools and methodologies to handle the complexity and heterogeneity of these datasets. Advancements in statistical methods and data integration techniques will be instrumental in overcoming these challenges and enabling comprehensive analyses of multi-ancestry data. The availability of longitudinal omics data across tissues would enable the analysis and prediction of health and disease patterns throughout life but would be challenging to achieve.

By addressing the gap of ancestrally diverse molecular data and advancing analytical methods, we can come one step closer to unlocking the full potential of genomics for health and pave the way for equity across populations in personalized medicine and precision therapeutics.
